# Endothelial dysfunction in renal arcuate arteries of obese Zucker rats: The roles of nitric oxide, endothelium-derived hyperpolarizing factors, and calcium-activated K^+^ channels

**DOI:** 10.1371/journal.pone.0183124

**Published:** 2017-08-17

**Authors:** Dandan Yin, Qianchen Wang, Xun Zhou, Ying Li

**Affiliations:** 1 Department of Nephrology, the Second Xiangya Hospital, Central South University, Key Laboratory of Kidney Disease and Blood Purification in Hunan, Changsha, China; 2 Department of Cardiovascular Medicine, Xiangya Hospital, Central South University, Changsha, China; Max Delbruck Centrum fur Molekulare Medizin Berlin Buch, GERMANY

## Abstract

The roles of nitric oxide (NO), endothelium-derived hyperpolarizing factors (EDHF), and calcium-activated K^+^ (K_Ca_) channels in diabetes-associated endothelial dysfunction of small renal arteries are not clear. The present study investigated acetylcholine (ACh)-induced vasorelaxation of renal arcuate arteries from obese Zucker (OZ) rats at different diabetes durations, and the relative contribution of NO, EDHF, and K_Ca_ channels to the endothelial dysfunction. OZ rats of 7 weeks (prediabetic stage), 12 weeks (early diabetic stage), and 20 weeks (late diabetic stage), and time-matched lean control rats, were studied. Segments of arcuate arteries (130 to 180 μm) were isolated, cannulated and pressurized. Vascular endothelial functions were tested using ACh-induced vasodilation. Our experiments demonstrated: (1) ACh-elicited vasodilation was impaired in OZ rats of 20 weeks, but not in rats of 7 and 12 weeks; (2) inhibition of NO or EDHF (contributed by epoxyeicosatrienoic acids [EETs]) production significantly decreased ACh-induced vasodilation in both lean and OZ rats of 20 weeks. The reduction of ACh-induced vasodilation by inhibition of NO or EDHF formation was less in OZ rats, as compared to lean rats; and (3) inhibition of K_Ca_ channels markedly reduced ACh-induced vasodilation in lean control rats, but not in OZ rats of 20 weeks. Our observations indicated that endothelium-dependent vasodilation in renal arcuate arteries is impaired in diabetes mellitus; NO and EDHF, mainly EETs, dominate the ACh-induced vasodilation in renal arcuate arteries; the contribution of NO and EETs is impaired in diabetic rats; K_Ca_ channels are involved in ACh-induced vasodilation; and the activity of K_Ca_ channels is downregulated in diabetes mellitus.

## Introduction

Endothelial dysfunction plays a key role in the pathogenesis of diabetic vascular complications, which account for most of the morbidity and mortality [1]. Substantial clinical and experimental evidence demonstrated that impaired endothelium-dependent relaxation existed consistently in both conduit and resistance arteries of diabetes mellitus [2–4]. Endothelial cells control vascular tone by releasing several relaxing factors, including nitric oxide (NO), endothelium-derived hyperpolarizing factors (EDHF), and prostacyclin. NO, derived from endothelial NO synthase (eNOS), is the principal mediator of acetylcholine (ACh)-induced, endothelium-dependent, relaxation in large conduit arteries. However, in small resistance arteries, including those of mesenteric, renal, and coronary circulation, EDHF is of increasingly greater significance in agonist-elicited vasorelaxation with decreasing vessel diameter [5–8]. A fundamental mechanism of vasodilation in small arteries is hyperpolarization through various types of potassium channels opening, and calcium-activated K^+^ channels (K_Ca_) are a key effector in control of endothelium-dependent EDHF-evoked vasorelaxation [6, 8–10].

Endothelium-dependent relaxation is an important regulatory mechanism in renal vessels. eNOS is typically expressed in endothelial cells along the renal vascular tree [11]. Inhibition of NO synthase (NOS) significantly reduced endothelium-dependent vasodilation in renal vessel beds [12]. Epoxyeicosatrienoic acids (EETs), generated from arachidonic acids by cytochrome P-450 (CYP) epoxygenase, have been identified as EDHFs in modulating vascular tone in renal vessels, and the contribution of EDHF to ACh-induced vasodilation was blocked by the inhibition of the K_Ca_ channel [7, 13]. The kidney is the organ typically responsible for microvascular complications of diabetes. Endothelial dysfunction in small renal vessels contributes to diabetic nephropathy, which is the most frequent cause of end-stage renal disease [14]. Moreover, the impaired contribution of NO or EDHF to endothelium-dependent vasodilation was demonstrated in renal arteries of rats with type 2 diabetes [15–18]. These findings raise the possibility that, in renal arterioles of type 2 diabetic rats, endothelial dysfunction results from the compromised function of NO, EDHF, and K_Ca_ channels. However, sporadic studies at some finite point in time have reported different findings [17, 19, 20]. Unaltered endothelium-dependent vasodilation [17], and no decreased release of NO [19, 20] has been shown in renal vessels of type 2 diabetic rats. The roles of NO, EDHF and, in particular, K_Ca_ channels in diabetes-associated endothelial dysfunction in renal small arteries are not clear.

Obese Zucker (OZ) rats have been widely used as a type 2 diabetes model. They develop obesity, hyperlipidemia, insulin resistance, and hyperglycemia, which result from a recessive mutation of the leptin receptor gene. Therefore, in the present study, OZ rats at different diabetic stages were used to examine ACh-induced vasorelaxation in renal arcuate arteries, and to investigate the contributions of NO, EDHF, and, particularly, K_Ca_ channels to endothelial dysfunction at the late stage of this animal model.

## Materials and methods

### Animals and vessel preparation

Male lean (*fa/+*) and OZ (*fa/fa*) rats were purchased from the Animal Center of Shanghai Institutes for Biological Sciences (Shanghai, China). The animals were housed in the Laboratory Animal Care Facility and the experiments were approved by the China Central South University Advisory Committee for Animal Resources. Rats of 7 weeks (prediabetic stage), 12 weeks (early diabetic stage), and 20 weeks (late diabetic stage), were used in the present study. Rats were anesthetized intraperitoneally with pentobarbital sodium (50 mg/kg). The kidneys were removed and placed in a cooling plate containing cold (0–4°C) 3-(N-morpholino) propanesulfonic acid (MOPS) buffered physiological saline solution (see below) containing 1% bovine serum albumin [8]. The kidneys were decapsulated and cut longitudinally. Segments of arcuate arteries were dissected, isolated, and cleared of adhering tubule and connective tissue, then were transferred to an organ bath (2.5 ml volume), and mounted on the stage of an inverted video microscope (Zeiss 100TV). To fit the glass cannulas, vessels with a maximum internal diameter from 130 to 180 μm were chosen. Arterial segments were cannulated at both ends onto glass micropipettes and secured, and the lumen of the vessel was filled with MOPS-buffered solution containing 1% albumin. The transmural pressure was set at 60 mmHg and continuously monitored. Neither transluminal flow nor oxygenation was applied to the cannulated vessels. The internal diameter of the vessels was recorded by a computerized diameter tracking system (Diamtrak, Montech Pty Ltd., Australia).

### Experimental procedures

After cannulation, the vessel diameter was measured at 60 mm Hg (maximum diameter [Dmax]). Then, the organ bath was heated from room temperature (~ 26°C) to 37.5°C and superfused continuously with a MOPS-buffered physiological saline solution (37.5°C, PH 7.3) of the following composition (in mmol/l): 144 NaCl, 3 KCl, 2.5 CaCl_2_, 1.4 MgSO_4_, 2.0 pyruvate, 5.0 glucose, 0.02 ethylenediaminetetraacetic acid (EDTA), and 2.0 MOPS, 1.21 NaH_2_PO_4_. To observe ACh-induced vasodilation after 30-minute equilibration in the bath, cannulated arcuate arteries were exposed to 1 μmol/l phenylephrine (PE) to produce a 50–60% contraction of maximum diameter; if not, PE was increased to 5μmol/l. When the vessel diameter is at a stable value it is considered to be in the steady PE-preconstriction state. After that, increasing doses of ACh were applied to incubation bath to elicit vasorelaxation. The vessel internal diameter was recorded once the vessel reached a steady state.

To investigate the possible mechanisms and alterations of ACh-induced vasodilation in diabetic renal arteries, various enzyme inhibitors and K^+^ channel blockers were applied at the following concentrations [21–23]: Nomega-nitro-L-arginine (L-NNA, 10 μmol/l) to inhibit NOS; indomethacin (10 μmol/l) to inhibit cyclooxygenase (COX); 6-(2-propargyloxyphenyl)hexanoic acid (PPOH, 10 μmol/l) or 17-octadecynoic acid (17-ODYA; 10 μmol/l) to inhibit the CYP epoxygenase; charybdotoxin (1 μmol/l) and apamin (1 μmol/l) to block K_Ca_ channels; glibenclamide (K_ATP_ inhibitor; 30 μmol/l); and barium chloride (K_ir_ channel inhibitor; 30 μmol/l). ACh-induced vessel diameter changes were recorded before and 30 minutes after incubation with these inhibitors. NS 1619 (1, 10, 50 and 100 μmol/l) was used to further test K_Ca_ channel activity. In addition, sodium nitroprusside (SNP) was used to investigate the function of smooth muscle. All chemicals were obtained from Sigma, St. Louis, U.S.A. The agents were dissolved in distilled water or DMSO, and the final concentration of DMSO in the working solution was less than 0.1% (vol/vol), at which no significant effect has been observed on vessel tone [24, 25].

### Data collection and statistical analysis

All data were presented as mean ± SD. Vessel diameter changes were presented as percentage (%) of dilation of the preconstriction, calculated as follows: % of vasodilation = [(Dagonist-Dbase)/(Dmax-Dbase)]x100, where Dmax was the diameter of the vessel at room temperature when the vessels were exposed to the pressure of 60 mm Hg, Dbase was the vessel diameter at steady constriction state induced by PE before ACh stimulation, and Dagonist was the diameter of the vessel after ACh stimulation. The maximum dilation was represented as 100%, and baseline diameter was 0%. Comparisons were made with the use of paired Student’s t-test or ANOVA with a post hoc Bonferroni test, as appropriate. The acceptable level of significance was defined as *P*<0.05.

## Results

### Body weights and blood glucose levels

**Fig 1:**
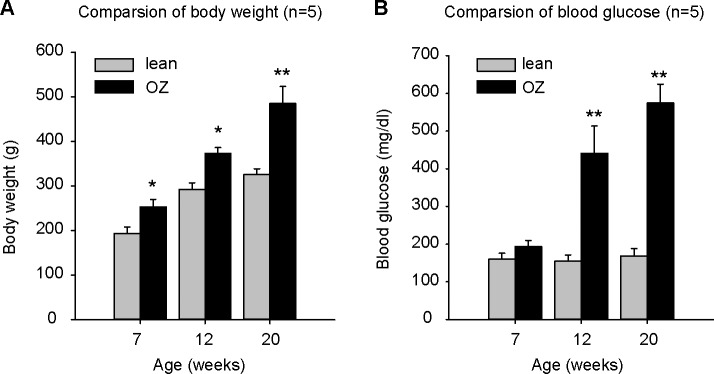
**Body weight (A) and blood glucose (B) data for each age-matched lean and obese Zucker (OZ) rats (n = 5)**. Body weight of OZ rats was greater than that in lean control rats (A). Blood glucose level significantly increased as expected in OZ rats at ages of 12 and 20 weeks (B). ** P<0.001, *P<0.05.

Body weight and blood glucose data for each age-matched lean and OZ rats are presented in Fig 1. Body weight was increased as the age of rats increased, but body weight of each group of OZ rats was greater than that of the lean rats (484.8±38.62 g compared to 325.56±13.00 g at 20 weeks) (Fig 1A). As expected, blood glucose levels were significantly increased in OZ rats at 12 weeks (440.6±73.04 mg/dl in OZ rats; 154.6±16.50 mg/dl in lean rats) and 20 weeks (574.2±50.63 mg/dl in OZ rats; 168.6±19.74 mg/dl in lean rats) (Fig 1B).

### Vascular responses to ACh in renal arcuate arteries of OZ rats at different diabetic stages

**Fig 2:**
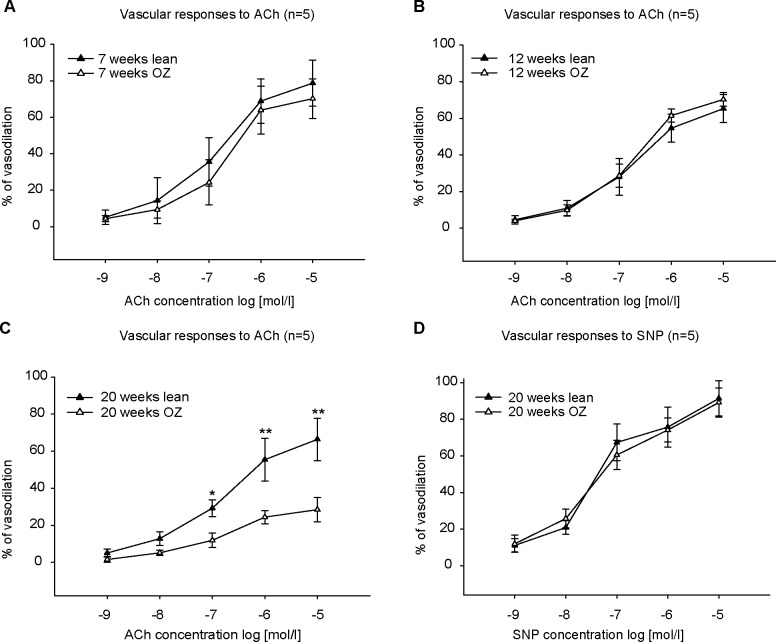
Concentration-response curves to acetylcholine (ACh) in phenylephrine (PE)-preconstricted renal arcuate arteries (n = 5). ACh-induced vasodilation in a concentration-dependent manner. The vasodilation in obese Zucker (OZ) rats was not different from lean control rats at the ages of 7 (A) and 12 (B) weeks. However, it was significantly decreased in vessels of OZ rats at the age of 20 weeks (C). Sodium nitroprusside (SNP) elicited a similar vasodilation in both OZ and lean rats of 20 weeks (D). ** P<0.001, *P<0.05.

ACh induced a concentration-dependent vasorelaxation in both lean and OZ rats of 7 weeks, 12 weeks, and 20 weeks (Fig 2). Removal of the endothelium by passing 1 ml air bubble through the vessel lumen abolished the vascular dilative responses (data not shown), which supports the assertion that ACh-induced vasorelaxation is endothelium-dependent in rat arcuate arteries. There was no significant difference of ACh-induced vasodilation between lean control and OZ rats at the ages of 7 (Fig 2A) and 12 weeks (Fig 2B). But the dilation was significantly reduced in OZ rats at 20 weeks of age (late diabetic stage) (Fig 2C). The dilation induced by 10 μmol/l ACh was only 28.42±6.53% in OZ rats compared to 66.37±11.44% in lean rats of 20 weeks. Since SNP is an endothelium-independent vasodilator, we then used SNP to test smooth muscle function. SNP elicited a similar vasodilation in both OZ and lean rats of 20 weeks (Fig 2D). Based on the impaired vasodilation results seen here, all the following experiments were conducted on renal arteries isolated from rats at the age of 20 weeks.

### The contribution of NO and EDHF to ACh-elicited vasodilation in renal arcuate arteries of late diabetic OZ rats.

**Fig 3:**
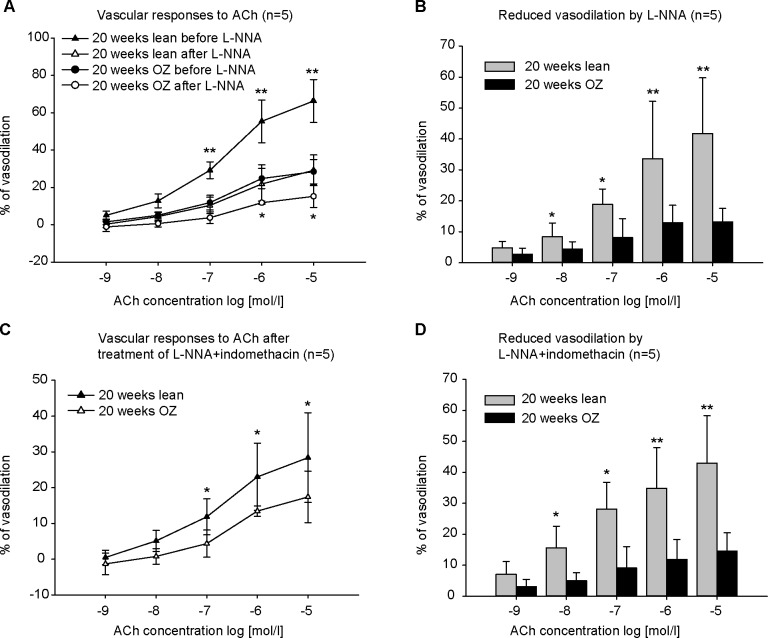
Concentration-response curves to acetylcholine (ACh) before/after incubation with L-NNA (10 μmol/l) or L-NNA plus indomethacin (10 μmol/l) in phenylephrine (PE)-preconstricted renal arteries (n = 5). L-NNA incubation resulted in a significant reduction in vasodilation in response to ACh in obese Zucker (OZ) rats and lean control rats (A), but the reduction was less in OZ rats than in lean rats (B). Combined treatment with L-NNA plus indomethacin did not further enhance the inhibitory effect of L-NNA. The reduction by L-NNA and indomethacin (D) was similar to that by L-NNA alone (B). Fig (C) shows the contribution of EDHF to ACh-induced vasodilation after treatment of L-NNA and indomethacin. The EDHF-mediated vasodilation was decreased in OZ rats. ** P<0.001, *P<0.05.

The contribution of NO and COX-derived prostaglandins was assessed by L-NNA and indomethacin, respectively. Incubation of vessels with L-NNA had a significant inhibitory effect on vasodilation induced by ACh, in both lean and OZ rats (Fig 3A). However, vasodilation was much less reduced by L-NNA in OZ rats than in lean rats. The reduction induced by 10 μmol/l ACh after L-NNA was 13.21±4.38% in OZ rats compared to 41.71±18.06% in lean rats (Fig 3B). Treatment with indomethacin did not cause an obvious inhibition of ACh-induced vasodilation (data not shown). The contribution of EDHF was assessed by the combined inhibition of NOS and COX. Combined application of L-NNA and indomethacin did not further enhance the inhibitory effect of L-NNA. The reduction elicited by L-NNA plus indomethacin (Fig 3D) was similar to that induced by L-NNA alone (Fig 3B). However, after combined incubation with L-NNA and indomethacin, ACh-induced vasodilation in OZ rats was less than that in lean control rats. The vasodilation induced by 10 μmol/l ACh was 17.40±7.21% in OZ rats compared to 28.38±12.55% in lean rats after application of L-NNA plus indomethacin (Fig 3C).

### The contribution of EETs/EDHFs to ACh-induced vasodilation in renal arcuate arteries of late diabetic OZ rats

**Fig 4:**
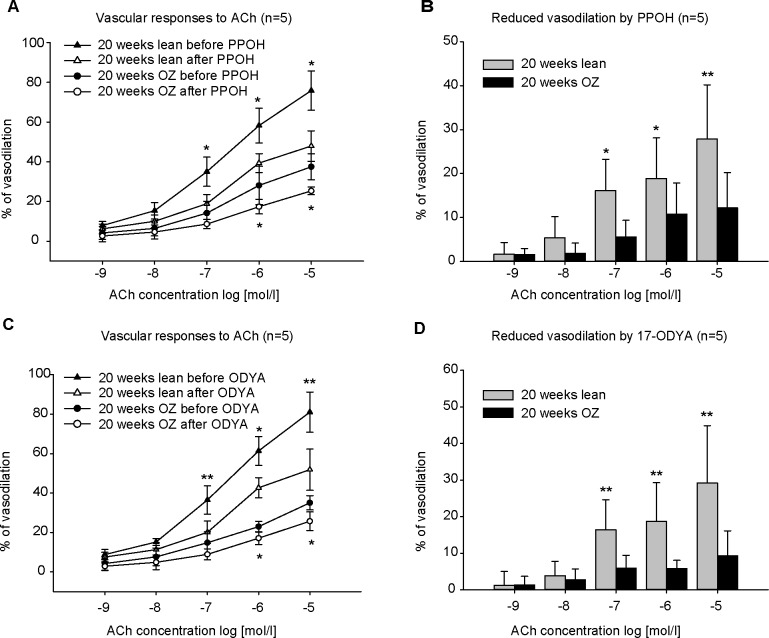
Concentration-response curves to acetylcholine (ACh) before/after inhibition of EET formation by PPOH (10 μmol/l) or 17-ODYA (10 μmol/l) in phenylephrine (PE)-preconstricted renal arteries (n = 5). Incubation with PPOH (A) or 17-ODYA (C) decreased ACh-induced vasodilation in lean and obese Zucker (OZ) rats. But the treatment with PPOH (B) or 17-ODYA (D) resulted in less reduction in OZ rats than in lean control rats. ** P<0.001, *P<0.05.

Since CYP epoxygenase is highly expressed in renal microvessels, and CYP epoxygenase metabolites, EETs, are identified as EDHFs in regulating renal vascular function, PPOH and 17-ODYA were used to inhibit epoxygenase activity. 10 μmol/L PPOH significantly decreased the ACh-induced vasodilation in renal arteries in both OZ and lean rats (Fig 4 A). The reduction evoked by PPOH in OZ rats was significantly less than that in lean rats. The reduction induced by 10 μmol/l ACh after PPOH in OZ rats was 12.16±8.05% compared to 27.88±12.33% in lean rats (Fig 4B). Incubation of 10 μmol/l 17-ODYA had a similar inhibitory effect (Fig 4C and 4D). The reduced vasodilation by 10 μmol/l ACh after 17-ODYA was 9.34±6.77% in OZ rats compared to 29.19±15.69% in lean rats (Fig 4D).

### K_Ca_ channel responses to ACh in renal arcuate arteries of late diabetic OZ rats

**Fig 5:**
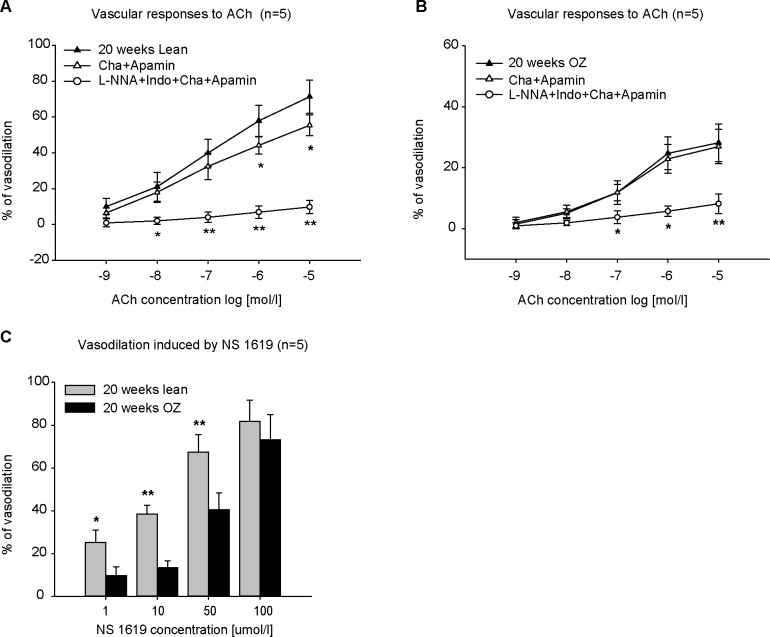
Concentration-response curves to acetylcholine (ACh) before/after incubation with charybdotoxin (Cha, 1 μmol/l) and apamin (1 μmol/l) in phenylephrine (PE)-preconstricted renal arcuate arteries (n = 5). The incubation with Cha and apamin decreased vasodilation in lean control rats (A), but no alternation was seen in obese Zucker (OZ) rats (B). Incubation with L-NNA, indomethacin (Indo), Cha, and apamin almost abolished vasodilation induced by ACh in both lean and OZ rats (A and B). NS 1619 elicited a concentration-dependent vasodilation in PE-preconstricted renal arcuate arteries (C). However, the vasodilation induced by 1, 10 and 50 μmol/l was decreased in OZ rats (C). ** P<0.001, *P<0.05.

EDHFs, in particular EETs, hyperpolarize smooth muscle through activation of K_Ca_ channels. We used charybdotoxin and apamin to block K_Ca_ channels. Incubation of vessels with charybdotoxin and apamin had an inhibitory effect on the vasodilation induced by ACh in lean rats (Fig 5A), but not in OZ rats (Fig 5B). A combined incubation with L-NNA, indomethacin, charybdotoxin and apamin almost abolished the ACh-induced vasodilation in both lean (Fig 5A) and OZ rats (Fig 5B). Blockade of K_**ATP**_ or K_**ir**_ channels by application of 30 μmol/l glibenclamide or 30 μmol/l barium chloride did not have any effect on the vasodilation induced by ACh (data not shown). To further test the activity of K_Ca_ channels, NS 1619 was used to activate them. NS 1619 elicited a dose-dependent vasodilation. 100 μmol/l NS 1619 almost evoked a full vasodilation in both lean and OZ rats. But the vasodilation induced by NS 1619 at low concentrations (less than 50 μmol/l) was significantly reduced in OZ rats than in lean rats (Fig 5C).

## Discussion

The present study investigated the influence of diabetes on the different stages and components of endothelium-dependent vasodilation in the renal arcuate arteries of OZ rats *in vitro*. The major findings are: (1) ACh-elicited vasodilation was impaired in late diabetic OZ rats at 20 weeks, but not in 7 week prediabetic or 12 week early diabetic rats; (2) Both NO and EDHF (contributed by EETs) mediated the ACh-elicited vasodilation in renal arcuate arteries of late diabetic OZ rats, and their contributions were reduced; and (3) K_Ca_ channels were involved in the ACh-elicited vasodilation and their function was impaired in late diabetic OZ rats.

Diabetes mellitus is a group of chronic metabolic disorders characterized not only by hyperglycemia, protein and lipid metabolism disorder, but also by macro- and micro-angiopathic vascular complications resulting from endothelial dysfunction, inflammation, apoptosis, and so on [1, 26]. Endothelial dysfunction is partially described as the impairment of endothelium-dependent relaxation. Few studies have examined the temporal nature of the onset of impaired endothelial-dependent dilation in renal vessels from animal models of type 2 diabetes. Gealekman et al. [17] found ACh-elicited vasodilation of renal interlobar arteries was significantly attenuated in 22-week-old OZ rats, but unimpaired in 8-week-old OZ rats. In our study, the ACh-induced vasodilation in renal arcuate arteries was investigated in OZ rats at different diabetic stages. ACh elicited a concentration-dependent vasodilation in pressurized renal arcuate arteries in vitro. Compared to the age-matched lean control rats, no difference was demonstrated in the prediabetic (7 weeks) and early diabetic (12 weeks) stages. But at the age of 20 weeks, ACh-induced vasodilation was significantly reduced in OZ rats. Our current observation, in agreement with the above report, revealed the importance of disease duration or the severity of diabetes in the development of vascular endothelial dysfunction.

Reduced eNOS activity and NO production, or decreased NO bioavailability, are some of the most important mechanisms of endothelial dysfunction in diabetes mellitus. But there are different observations in renal vasculature. Enhanced [19], normal [27], and impaired [18] NO production and/or activity have been demonstrated in rodent models of type 2 diabetes. In the current study, NO appeared to dominate the vasodilatory responses to ACh in renal resistance arteries in both OZ diabetic and lean rats at 20 weeks of age. Inhibition of NOS markedly decreased ACh-induced vasodilation in these animals. The reduction was less in OZ rats than in lean rats, confirming impaired NO production or activity in the renal vessels of diabetic rats.

EETs have been identified as EDHFs in the kidney [13, 15, 21]. However, few studies have investigated the role of EDHF in renal arteries of diabetes mellitus, especially in those of type 2 diabetes. Zhao et al. [15] reported that endothelium-dependent EDHF-evoked dilation was impaired in the renal preglomerular vasculature of 20-week-old OZ rats. In our preparation, a portion of endothelium-dependent vasodilation induced by ACh was still observed in both lean rats and OZ rats after the combined inhibition of NOS and COX, but vasodilation was decreased in OZ rats after blocking NOS and COX. Consistent with the previous study, our results indicated that EDHF played an important role in mediating ACh-induced vasodilation in renal arcuate arteries, and the contribution of EDHF was impaired under diabetic conditions.

To further investigate the EDHF identity, PPOH, a specific CYP epoxygenase inhibitor, was tested in our preparation. Treatment with PPOH reduced ACh-elicited vasodilation in both OZ rats and lean control rats at 20 weeks of age. The reduction by PPOH was less in OZ diabetic rats. The results were confirmed by use of another irreversible CYP epoxygenase inhibitor, 17-ODYA. Our observations indicated that CYP epoxygenase metabolites, namely EETs, contributed to ACh-induced vasodilation in renal arteries, and this contribution was reduced in diabetic rats.

The opening of K_Ca_ channels on vascular smooth muscle cells, and the resulting hyperpolarization, is the major feature of EDHF-mediated vasorelaxation. Experiments using electrophysiological and pharmacological approaches have revealed functional K_Ca_ channels in renal microvessels [28, 29]. Blockage of K_Ca_ channels diminished the vasodilatory response to 11, 12-EET in renal microvessels [21]. However, the role of K_Ca_ channels in renal arteries in diabetes mellitus has not been thoroughly studied. It is known that diabetic endothelial status is associated with increased production of reactive oxygen species (ROS) [30]. Also, ROS, especially hydrogen peroxide (H_2_O_2_) and peroxynitrite (ONOO^-^), effectively inhibited K_Ca_ channel function [31, 32]. In the present study, inhibition of K_Ca_ channels with charybdotoxin and apamin decreased ACh-induced vasodilation in lean rats, but not in OZ diabetic rats at 20 weeks of age, which indicated that K_Ca_ channel activity was impaired in diabetic animals. This conclusion was further confirmed by the application of NS 1619, a specific K_Ca_ channel agonist. NS 1619 elicited-vasodilation was reduced in diabetic OZ rats compared to controls. Our current observation, in agreement with the findings reported in retinal vascular smooth muscle [33], supported the idea that the activity of K_Ca_ channels was downregulated in diabetes mellitus. In our preparation, inhibition of K_**ATP**_ and K_**ir**_ channels by glibenclamide and low concentrations of barium chloride did not elicit marked alteration of ACh-induced vasodilation, suggesting that K_**ATP**_ and K_**ir**_ channels play little or no role in ACh-induced vasodilation in renal arcuate arteries.

**Fig 6:**
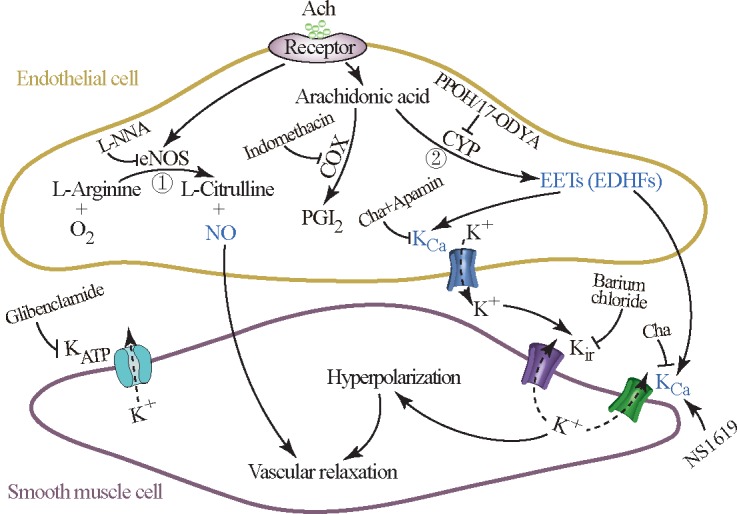
Roles of nitric oxide (NO), endothelium-derived hyperpolarizing factors (EDHF), and calcium-activated K^+^ (K_Ca_) channels in renal arcuate arteries. Acetylcholine (ACh) can induce endothelium-dependent vasodilation by the release of NO (①) and EDHF (②) from endothelial cells in renal arcuate arteries. NO is derived from endothelial NO synthase (eNOS), while prostacyclin (PGI_2_) and epoxyeicosatrienoic acids (EETs, as EDHFs) are generated from arachidonic acids by cyclooxygenase (COX), and cytochrome P-450 (CYP) epoxygenase, respectively. EETs facilitate the hyperpolarization of smooth muscle cells and vascular relaxation through K^+^ efflux mediated by the opening of K_Ca_ channels either in endothelial cells or in smooth muscle cells. The K^+^ released from K_Ca_ channels of endothelial cells into the subendothelial space (potential connective tissue space beneath the endothelium) subsequently actives inward rectifying K^+^ (K_ir_) channels on smooth muscle cells, while producing K^+^ efflux as well. In the present study, the contribution of NO and EDHF to endothelium vasodilation was impaired and the activity of K_Ca_ channels was downregulated in renal arcuate arteries of obese Zucker (OZ) rats at 20 weeks of age, but PGI_2_ had no effect on ACh-elicited vasodilation. Barium chloride, inhibitor of the K_ir_ channel; Charybdotoxin (Cha)+Apamin, inhibitor of the K_Ca_ channel; Glibenclamide, inhibitor of the K_ATP_ channel; Indomethacin, inhibitor of COX; L-NNA, inhibitor of eNOS; NS1619, agonist of the K_Ca_ channel; and PPOH or 17-ODYA, inhibitors of CYP epoxygenase.

In conclusion, the present study provided evidence supporting the hypothesis that NO, EDHF (mainly EETs), and K_Ca_ channels dominate ACh-induced vasodilation in renal arcuate arteries; furthermore, the contribution of NO and EDHF was impaired, and the activity of K_Ca_ channels was downregulated, in renal arcuate arteries of late diabetic OZ rats (Fig 6). This study provides a specific basis for evaluating the appropriate timing of pharmacological intervention to prevent endothelial dysfunction in renal microvessels under diabetic conditions. It also provides mechanistic basis for developing novel drugs to ameliorate endothelial dysfunction in renal small arteries in diabetes mellitus.
