# Correction: Endothelial dysfunction in renal arcuate arteries of obese Zucker rats: The roles of nitric oxide, endothelium-derived hyperpolarizing factors, and calcium-activated K^+^ channels

**DOI:** 10.1371/journal.pone.0204297

**Published:** 2018-09-28

**Authors:** The PLOS ONE Staff

The first and second authors’ names are spelled incorrectly. The correct names are: Dan-dan Yin and Qian-chen Wang. The correct citation is: Yin DD, Wang QC, Zhou X, Li Y (2017) Endothelial dysfunction in renal arcuate arteries of obese Zucker rats: The roles of nitric oxide, endothelium-derived hyperpolarizing factors, and calcium-activated K^+^ channels. PLoS ONE 12(8): e0183124. https://doi.org/10.1371/journal.pone.0183124
